# Determination of Diffusion Kinetics of Ketamine in Brain Tissue: Implications for *in vitro* Mechanistic Studies of Drug Actions

**DOI:** 10.3389/fnins.2021.678978

**Published:** 2021-07-01

**Authors:** Zachary Geiger, Brett VanVeller, Zarin Lopez, Abdel K. Harrata, Kathryn Battani, Lauren Wegman-Points, Li-Lian Yuan

**Affiliations:** ^1^Department of Physiology and Pharmacology, College of Osteopathic Medicine, Des Moines University, Des Moines, IA, United States; ^2^Department of Chemistry, Iowa State University, Ames, IA, United States

**Keywords:** ketamine, antidepressant, pharmacokinetics, brain, hippocampus slice, NMDA receptor, synaptic plasticity

## Abstract

Ketamine has been in use for over 50 years as a general anesthetic, acting primarily through blockade of *N*-methyl-D-aspartate receptors in the brain. Recent studies have demonstrated that ketamine also acts as a potent and rapid-acting antidepressant when administered at sub-anesthetic doses. However, the precise mechanism behind this effect remains unclear. We examined the diffusion properties of ketamine in brain tissue to determine their effects in *in vitro* studies related to the antidepressant action of ketamine. Brain slices from adult mice were exposed to artificial cerebrospinal fluid (aCSF) containing ∼17 μM ketamine HCl for varying amounts of time. The amount of ketamine within each slice was then measured by tandem high-performance liquid chromatography – mass spectrometry to characterize the diffusion of ketamine into brain tissue over time. We successfully modeled the diffusion of ketamine into brain tissue using a mono-exponential function with a time constant of τ = 6.59 min. This curve was then compared to a one-dimensional model of diffusion yielding a diffusion coefficient of approximately 0.12 cm^2^⋅s^–1^ for ketamine diffusing into brain tissue. The brain:aCSF partition coefficient for ketamine was determined to be approximately 2.76. Our results suggest that the diffusion properties of ketamine have a significant effect on drug concentrations achieved within brain tissue during *in vitro* experiments. This information is vital to determine the ketamine concentration necessary for *in vitro* slice preparation to accurately reflect *in vivo* doses responsible for its antidepressant actions.

## Introduction

Depression is a chronic and debilitating psychiatric illness affecting roughly 16% of the United States United States population. Current pharmacologic therapies targeting monoamine neurotransmission exhibit limited effectiveness, notably leaving ∼30% of patients with treatment-resistant depression (TRD) without relief. Even when effective, current treatments take 4–6 weeks for maximum effects, leaving depressive patients vulnerable to suicidal ideations. However, recent evidence has shown ketamine, a glutamate receptor antagonist, to be a highly effective antidepressant representing a breakthrough in the development of the next generation of rapid-acting antidepressants with success in patients with TRD. At sub-anesthetic doses, ketamine has shown promising results as a potent and fast-acting antidepressant (Krystal et al., 1994; Berman et al., 2000). These effects are particularly evident in patients with TRD and in patients with high risk of suicide (Mathew et al., 2012; Naughton et al., 2014). While it has long been used as general anesthetic acting primarily through blockade of *N*-methyl-D-aspartate (NMDA) receptors in the brain, ketamine exhibits a wide range of pharmacologic actions in a concentration dependent manner; its effects include hypnotic sedation, psychotogenic actions, profound analgesia, increased sympathetic tone, and attenuation of chronic pain (Sleigh et al., 2014).

Animal model studies have identified multiple potential mechanisms of action for ketamine’s antidepressant effects. These include rapid suppression of neuronal firing in the lateral habenula and activation of signaling cascades culminating in neurotrophic factor release in the prefrontal cortex (PFC) and hippocampus within a few hours, followed by a second wave of synaptic protein upregulation (Li et al., 2010; Autry et al., 2011; Yang et al., 2018). Together, these molecular events are thought to lead to rapid synaptogenesis and are responsible for the reversal of neural atrophy seen in rodent models (Li et al., 2010).

Previous investigations into the molecular and cellular mechanisms underlying ketamine’s antidepressant actions have primarily focused on its direct effect on NMDA receptors, often utilizing an *in vitro* setting with brain slices bathed in artificial cerebrospinal fluid (aCSF; Autry et al., 2011; Zanos et al., 2016; Suzuki et al., 2017; Yang et al., 2018). As stated earlier, ketamine causes a wide array of clinical responses in a concentration dependent manner, consequently even small deviations in concentration could have a large impact on the neuronal response. Ketamine is both hydrophilic and lipophilic, properties that will affect its movement when presented with the *in vitro* interface of aCSF and lipid-rich brain tissue, potentially contributing to an uneven distribution between these mediums. This selective partitioning of ketamine into brain tissue has a significant impact on the effective dose of ketamine the tissue receives and should be carefully considered when determining the amount of drug to add to the aCSF. When animals were intraperitoneally injected with ketamine at its antidepressant dose (10–25 mg/kg), the ketamine concentration achieved in brain tissue immediately/within a few minutes following injection was estimated to be 1.2–2.6 μg/g or 6–11 μM (Zou et al., 2009; Lilius et al., 2015; Zanos et al., 2016; Yang et al., 2018). To recapitulate this therapeutically relevant concentration in an *in vitro* setting ketamine’s partition coefficient between brain tissue and surrounding aCSF must be taken into consideration. To address this concern, we examined the diffusion kinetics of ketamine in brain tissue in order to determine the relevant concentrations of ketamine which must be applied to brain tissue *in vitro* to appropriately represent *in vivo* conditions.

## Methods

### Mouse Brain Slice Preparation

The use of animals for the studies described below was approved by the Des Moines University Institutional Animal Care and Use Committee. Transverse hippocampal slices were prepared from 2 to 4 months old male and female C57 BL6/J mice, following established procedures (Zhao et al., 2011). Briefly, mice were euthanized by cervical dislocation. Both hemispheres then were removed quickly and sliced to 300–350 μM thickness with a Vibratome with ice-cold cutting solution containing the following (in mM): 240 sucrose, 2.5 KCl, 1.25 NaH_2_PO_4_, 25 NaHCO_3_, 0.5 CaCl_2_, and 7 MgCl_2_, saturated with 95% O_2_/5% CO_2_ (freezing prior to use). Incubations were performed in aCSF (in mM): 125 NaCl, 2.5 KCl, 1.25 NaH_2_PO_4_, 25 NaHCO_3_, 2.0 CaCl_2_, 1.0 MgCl_2_, and 25 Dextrose.

### Ketamine Uptake and Tissue Extraction

Individual 300 μm-thick brain slices were prepared and placed into an incubation beaker containing 150–200 mL aCSF with 17.1 μM ketamine HCl. The solution was saturated and thereafter constantly bubbled with 95% O_2_/5% CO_2_. Air bubbling provided constant, gentle agitation to the solution. Slices rested on a single layer of mesh allowing ketamine to diffuse into tissue from both surfaces. At selected time points between 0.5 and 120 min a slice was removed from the solution, blotted to removed excessive liquid on the surface, and placed into a centrifuge tube containing 100 μL ddH_2_O. Each tubed was weighed before and after transfer of the brain tissue in order to calculate the weight of each slice. The slice was homogenized via pestle and centrifuged for 30 min at 4,000 × *g*. The supernatant was transferred to a clear tube and stored at −80°C until further analysis (Gredell et al., 2004).

### Chromatographic Separation and Analysis

The amount of ketamine present in each sample was quantified using the Agilent 1200 series tandem high-performance liquid chromatography coupled with the Agilent QTOF 6540 mass spectrometry (LCMS) equipped with the JetSream ESI ion source. All samples were injected in the same conditions using the Agilent 1200 series Autosampler. Samples were separated using an Agilent XDB-C18, 150 × 4.6 mm, 1.8 μm particle size Eclipse column. Mobile phase consisted of A: H_2_O,0.1% Formic Acid, B: Acetonitrile. Gradient conditions consisted of ramping solvent B from 30 to 40% in 2 min then to 100% in.5 min and maintained for 1.5 min at 100%. m/z 122 of protonated N-methylbenzylamine (internal standard) and m/z 238 of protonated Ketamine mass spec peaks were extracted from the total ion current, smoothed, and integrated.

### Computational Modeling

Diffusion properties of ketamine were modeled using a one-dimensional diffusion equation (Eq. 1). The geometry of a brain slice is such that a one-dimensional model is appropriate to represent ketamine diffusing into both sides of a brain slice (Chesney et al., 2003).

(1) $$Y=\sum_{j=1}^{\infty }\left(\frac{\left(-2\,{\left(-1\right)}^{j}\right)}{\left[\left(2\,j-1\right)\,/\,2\right]\,\pi }\,e^{-1\,{\left[\left(2\,j-1\right)\,\pi \,/\,2\right]}^{2}\,\tau }\,\cos\left[\left(\frac{2\,j-1}{2}\right)\,\pi \cdot n\right]\right)$$

where

(2) $$Y=\frac{C_{f}-C}{C_{f}}$$

(3) $$n=x\,/\,x_{0}$$

(4) $$\tau =\frac{D\cdot t}{x_{0}^{2}}$$

Equation 1 One-dimensional diffusion equation given in dimensionless terms of concentration (2), thickness (3), and time (4) with equilibrium concentration *C*_*f*_, concentration *C* at time *t*, depth *x*, slice thickness *x*_0_ (Since ketamine diffused into brain slices of 300 μm from both surfaces, effective slice thickness is 150 μm), and diffusion coefficient *D* (Gredell et al., 2004).

### Electrophysiological Recordings

Individual 350 μm-thick hippocampal slices were prepared and transferred to a recording chamber perfused with aCSF at 29∼30°C. Recording electrodes were placed in the stratum radiatum within CA1 and presynaptic stimulation was given at the Schaffer collaterals within, or directly adjacent to, the CA3/CA1 boundary. Signals were recorded using a Dagan amplifier BVC 700A (Dagan Corporation, Minneapolis, MN, United States). Noise was reduced via a faraday cage surrounding the recording setup and Humbug 50/60 Hz noise removing hardware (Quest Scientific, North Vancouver, BC, Canada). A 100 μs presynaptic stimulation pulse was evoked using 1 MΩ, bipolar, tungsten stimulating electrodes (FHC, Bowdoin, ME, United States) driven by A365R constant-current stimulus isolators (World Precision Instruments, Sarasota, FL, United States).

To isolate field excitatory synaptic potentials (fEPSPs) mediated by NMDA receptor-mediated, we first recorded total fEPSPs at the maximal stimulation without inducing population spikes in normal aCSF. 10 μM CNQX in 0.1 mM Mg^2+^-containing aCSF was perfused to block AMPA receptors. Stimulus width (100 μs) was then doubled to maximize the chance of effectively activating NMDA receptors. Responses recorded under this condition were confirmed to be solely mediated NMDA receptors by application of their antagonist APV (20 μM). Stable baseline responses were established for 20 min prior to application of ketamine.

In order to monitor ketamine induced changes in AMPA receptor-mediated fEPSPs, the stimulus intensity was adjusted so the fEPSP slope was 40–50% of the maximum and test pulses delivered every 30 s. After a minimum of 10 min recording stable baseline fEPSP responses, ketamine, at varying concentrations (2–20 μM), was applied to the bath for 20 min during which no stimulus was applied. fEPSPs were recorded for another 50–60 min following ketamine washout. The slope of fEPSP was quantified to reflect synaptic strength. The magnitude of synaptic potentiation was quantified by normalizing fEPSP slope to baseline pre-ketamine responses.

### Drugs

APV, CNQX, D-serine, and Ketamine hydrochloride were purchased from Tocris Bioscience (Bristol, United Kingdom). [Selective antagonists for AMPA and NMDA receptors, respectively, CNQX (6-cyano-7-nitroquinoxaline-2,3-dione disodium salt hydrate) and APV (D(-)-2-Amino-5-phosphonopentanoic acid)].

### Data Analysis and Statistics

Data were analyzed and plotted using Origin (Microcal) and Igor (WaveMetrics). Fits to mono-exponential functions were calculated using non-linear least square minimization. Significance (*p* < 0.05) was determined by one-way ANOVA, followed by *post hoc* Tukey tests. Error bars represent SEM.

## Results

**FIGURE 1:**
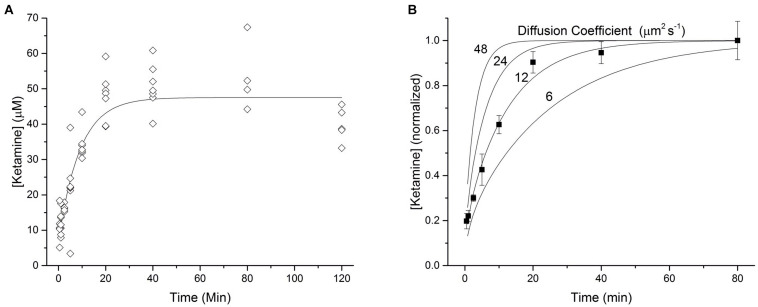
Kinetics of ketamine diffusion into brain tissue. **(A)** 300 micron thick brain slices were incubated in a 17.1 μM solution of ketamine HCl dispersed in ACSF for various lengths of time ranging from 0.5 to 120 min (*n* = 4–7 slices for each time point, *N* = 6 mice). Each point represents the average tissue concentration of ketamine within a single brain slice. The line of best fit represents a mono-exponential function with a time constant of τ = 6.59 min. **(B)** A one-dimensional model of diffusion was used to generate four curves representing the diffusion of a hypothetical drug, over a range of diffusion coefficients, into both the top and bottom surfaces of a 300 micron thick brain slice. Average tissue concentrations of ketamine, normalized to the average concentration observed at 80 min, were plotted against these curves to estimate the diffusion coefficient of ketamine in brain tissue. Each point represents the mean value at each time point from A bounded by the corresponding standard error of the mean.

In order to characterize the diffusion kinetics of ketamine into brain tissue, 300 μm hippocampal slices were incubated in aCSF containing 17.1 μM ketamine hydrochloride with gentle agitation for various lengths of time between 0.5 and 120 min. The absolute amount of ketamine extracted from each hippocampal slice was determined via analysis of tandem high-performance liquid chromatography – mass spectrometry. Based on the amount of ketamine determined in each slice, the mass of individual slices, the density of brain tissue (1.04 g/cm^3^; Weaver et al., 2001), and the molecular weight of ketamine HCl (274.2 g/mol), an average tissue concentration was calculated and reported (Figure 1). As illustrated in Figure 1A, ketamine quickly partitioned into hippocampal slices and reached an equilibrium state. The results of these measurements were used to construct a model of ketamine uptake into brain tissue. The equilibration time constant was determined to be τ = 6.59 min by fitting the data with a mono-exponential function (solid line, Figure 1A). An orthogonal distance regression algorithm was utilized to yield a fit with an adjusted coefficient of determination of *R*^2^ = 0.9902.

On the basis of the line with the best fit, the equilibrium tissue concentration of ketamine was determined to be 47.2 μM. This value was then divided by 17.1, the known ketamine concentration in the surrounding aCSF, yielding a brain:aCSF partition coefficient for ketamine of approximately 2.76. Translated as, for any given amount of ketamine in the aCSF, the submerged tissue will experience almost 3 times that concentration. As shown in Figure 1, ketamine concentrations declined below the equilibrium concentration after 120 min of incubation, possibly due to ketamine metabolism *in vitro* or changes in tissue properties resulting from the extended incubation times. Therefore, we chose 80 min as the final time point when determining the diffusion coefficient for ketamine in brain tissue.

Using a one-dimensional model of diffusion (Eq. 1, Gredell et al., 2004), *expected* tissue concentrations of ketamine corresponding to a range of diffusion coefficients (6–48 μm^2^ s^–1^ were plotted in Figure 1B. Average *measured* tissue concentrations of ketamine (from Figure 1A) were normalized to the concentration at 80 min and plotted against diffusion curves derived from the computational model. By comparing the ketamine data to these hypothetical curves we can approximate the diffusion coefficient of ketamine in brain tissue to be about 12 μm^2^ s^–1^.

**FIGURE 2:**
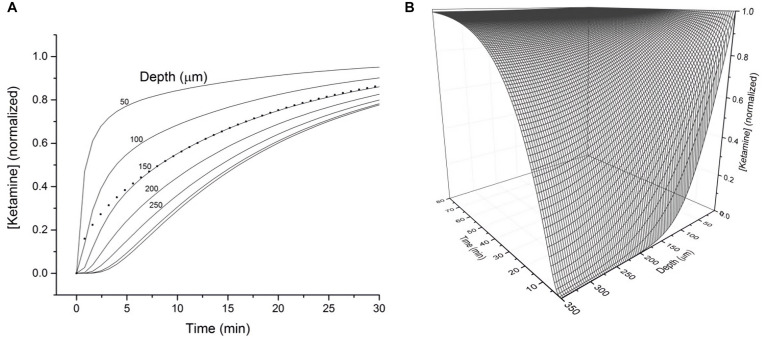
**(A)** Calculated tissue concentration of ketamine (diffusion coefficient *D* = 12 μm^2^ s^–1^) as function of depth and time within a 350 micron thick brain slice exposed on the top surface to a solution of ketamine in ACSF. The dotted line represents the average concentration of ketamine within the entire 350 micron thick slice as a function of time. **(B)** 3-D representation of the diffusion kinetics as a function of time and depth.

To assess how the diffusion characteristics of ketamine affect the tissue concentrations of the drug under typical electrophysiology recording conditions, the estimated ketamine diffusion coefficient (D) of 12 μm^2^ s^–1^ was incorporated into a model. Typical brain slice recordings utilize tissue blocks of 300–400 μm in thickness placed in a recording chamber, allowing one surface of the tissue block to be in direct contact with aCSF. Hence, this model sought to demonstrate the concentration of ketamine as a function of depth and time within a 350 μm thick brain slice exposed to a solution of ketamine in aCSF on one surface (Figure 2A). The depth-concentration profile modeled this way revealed a significant difference between the slice surface and more interior portions of the slice. Specifically, at the depth of 50 μm where visualized patch recordings are usually performed, the tissue concentration will have almost reached its equilibrium state within 10 min. Whereas at the depth of 150 μm where extracellular field recordings are typically performed, the tissue will have only reached 50% of its final equilibrium concentration within a similar time frame. Tissue depths of more than 150 μm will take even longer time to reach the final concentration. A three-dimension representation of the same modeling results in shown in Figure 2B.

**FIGURE 3:**
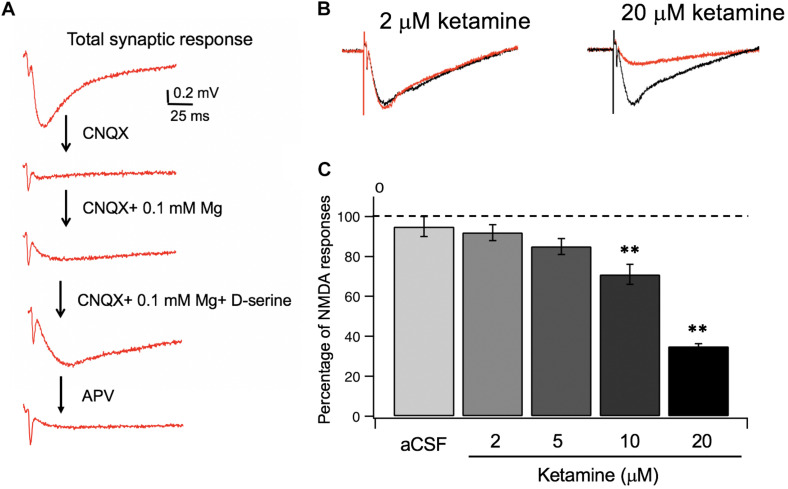
Ketamine’s concentration-dependent effects on synaptic NMDA responses. **(A)** Pharmacological isolation of synaptic NMDA responses in hippocampal slices. Field EPSPs (fEPSP) recorded in normal aCSF are primarily mediated by AMPA receptors **(a)**, which were completely blocked by CNQX, an AMPA receptor antagonist **(b)**. Wash-in of aCSF containing 0.1 mM Mg2 + unblocks NMDAR, resulting in a slow and small voltage changes in postsynaptic membranes **(c)**. Lastly, addition of D-serine, the endogenous NMDAR agonist, resulted in an enhanced synaptic NMDA response **(d)**, which was completely blocked by APV, an NMDA receptor antagonist **(e)**. **(B)** Representative traces of synaptic NMDA responses in response to 2 and 20 μM ketamine. **(C)** Summary data of dose dependent ketamine blockade of synaptic NMDA responses quantified by total charge transfer (i.e., response area) and normalized to pre-ketamine baseline responses. The NMDA response was significantly reduced by 10, and 20 μM of ketamine (**p < 0.01) but not altered at 2 or 5 μM (*p* > 0.05; One-way ANOVA followed by *post hoc* Tukey tests).

Utilizing the information on ketamine’s partition coefficient and other diffusion properties, we examined the dose-response relationship between ketamine and its target, NMDA receptors in glutamatergic synapses of mouse hippocampus. In order to isolate NMDA receptor-mediated responses, we first evoked a synaptic response at the Schaffer collateral – CA1 pathway in the hippocampal slice and then used a series of pharmacological manipulations to isolate and confirm the specificity of the response (Figure 3A). After obtaining a robust and stable NMDA response, we then tested how synaptic NMDA responses responded to various concentrations of ketamine. Because of the slow kinetics of NMDAR-mediated fEPSP, we examined total charge transfer, as quantified by area covered by fEPSP, which is more sensitive to changes than fEPSP amplitude (Welch et al., 2007). The NMDA responses were then normalized to pre-ketamine baseline response as the percentage of ketamine blockade. As shown in Figures 3B,C, ketamine exerted a significant blocking effect on NMDA receptor-mediated responses [*F*(4, 20) = 33.78, *p* < 0.0001; one-way ANOVA]. Tukey *post hoc* tests revealed that ketamine at 10 and 20 μM blocked the responses significantly by ∼30 and ∼65%, respectively, (*n*/*N* of slices/mice = 5/5, 4/4; *p* < 0.01, *p* < 0.01), in comparison with the aCSF control (*n*/*N* = 5/5). It also produced a small (∼10%) but insignificant reduction of the NMDA synaptic response at 5 μM (*n*/*N* = 6/6, *p* > 0.05). The clinically relevant ketamine dose of 2 μM was also not effective either in blocking the response (*n*/*N* = 5/5, *p* > 0.05).

**FIGURE 4:**
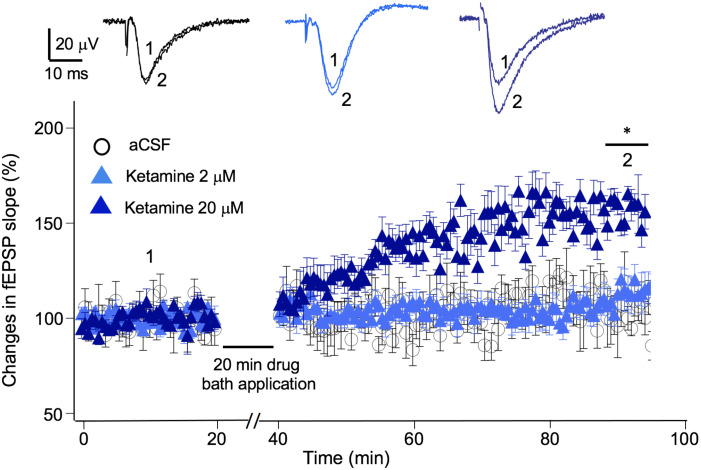
Ketamine’s concentration-dependent effect on synaptic plasticity. Time course and magnitude of synaptic plasticity in hippocampal CA1 region induced by bath applied ketamine for 20 min at concentrations of 0 (*p* > 0.05), 2 μM (*p* > 0.05), and 20 μM (*p < 0.05; One-way ANOVA followed by *post hoc* Tukey tests). The magnitude of synaptic potentiation was quantified by normalizing fEPSP slope to baseline pre-ketamine responses. Inset shows representative traces of fEPSPs before and after ketamine application.

In addition to exerting a blocking effect on NMDA receptors, ketamine is reported to enhance AMPA receptor-mediated synaptic responses (Duman, 2014). Previous studies suggest, this LTP-like effect can be achieved in the presence or in the absence of electrical stimulation, e.g., no synaptic responses evoked during ketamine application (Autry et al., 2011; Aguilar-Valles et al., 2020). We used the “no-stimulation protocol” to assess the effect of a range of ketamine concentrations (2–20 μM) on synaptic strength. The magnitude of synaptic potentiation was quantified by normalizing fEPSP slope to baseline pre-ketamine responses. As shown in Figure 4, ketamine exerted a significant potentiating effect on fEPSPs [*F*(2, 12) = 5.94, *p* < 0.05; one-way ANOVA]. Tukey *post hoc* tests revealed that bath application of 20 μM ketamine for 20 min induced a significant increase in fEPSP slope in 50–60 min in comparison to the aCSF control (20 μM ketamine 156 ± 7.2%, *p* < 0.05; *n*/*N* = 6/5; aCSF: 102 ± 7.8%, *n*/*N* = 5/5), while 2 μM ketamine did not (107 ± 9.7%, *n*/*N* = 6/5).

## Discussion

The partition coefficient is defined as the concentration ratio of a compound between two media at equilibrium. Ketamine, a chemical derivative of phencyclidine, is highly soluble in both lipids and in water. Here we examined the partition coefficient of the compound ketamine within the context of a brain slice submerged in aCSF, a preparation commonly used for electrophysiological recordings. While derived from a specific experimental condition, the partition coefficient is a constant, independent of slice thickness and ketamine concentration within most physiological experimental setup parameters. Our results suggest that in lipid-rich brain tissue, ketamine concentrations quickly rise to about 2.8 times the concentration seen in the surrounding aqueous aCSF. This ratio is consistent with previously reported partition coefficient of 2.2 as determined by gas chromatography (Cohen and Trevor, 1974). This is significant because, in an *in vitro* setting in which brain slices are bathed with ketamine-containing aCSF, the partitioning of ketamine selectively into brain tissue has a significant impact on the effective dose of ketamine received by the brain tissue.

The ketamine concentration achieved in the rodent brain following the administration of a subanesthetic antidepressant dose of ketamine (10 mg/kg via IP) has been estimated as 5–6 μM. This measurement has been consistent across different brain regions among multiple studies (Zou et al., 2009; Lilius et al., 2015; Zanos et al., 2016; Yang et al., 2018). To achieve this concentration in an *in vitro* setting we must divide the tissue concentration found *in vivo* by the partition coefficient to reach the appropriate concentration to add in the aCSF. Based on our estimate of the ketamine partition coefficient between brain tissue and aCSF, to achieve a 5–6 μM concentration in the tissue, the aCSF concentration of ketamine should be ∼2–2.5 μM.

This increased partitioning of ketamine in the brain tissue is important not only when designing new experimental paradigms, but also when interpreting results from prior *in vitro* studies. Taking into account the ketamine brain:aCSF partition coefficient calculated here, one must consider the reported concentrations of ketamine in the aCSF represent ∼30–40% of the concentrations of ketamine actually achieved within the brain tissue. Previous electrophysiological investigations examining ketamine’s effects at the synaptic receptor level have administered anywhere from 5 to 50 μM ketamine to brain slices via aCSF. Factoring in our experimentally determined partition coefficient, that represents dosages ranging from ∼15 to 150 μM, far exceeding the concentration thought to be relevant to ketamine’s antidepressant effects (Autry et al., 2011; Suzuki et al., 2017; Yang et al., 2018; Aguilar-Valles et al., 2020). Due to the concentration-dependent nature of ketamine’s spectrum of actions, unintended overdosing of ketamine could lead to effects which are vastly different from those responsible for ketamine’s antidepressant actions.

While both the time constant and diffusion coefficient suggest ketamine diffusion into brain tissue is a relatively rapid process, tissue depth in an *in vitro* setting represents a significant factor in determining the amount of time for ketamine to reach its final equilibrium concentration. Based on the modeled depth-concentration profile (Figure 1B), experimental results derived from different recording techniques (visualized patch recordings targeting neurons on the surface vs. extracellular recordings collecting responses from neurons at depth of 150 μm) should consider these variables when determining the ketamine sensitivity of NMDA receptors.

Ketamine, and its various metabolites, interact with a broad range of targets. Currently, the central goal of ketamine research focuses on delineating the dissociative pathways from the antidepressant ones. Several lines of evidence have contributed to an ongoing debate on ketamine’s antidepressant action being NMDA receptor dependent or independent. A recent paper identified (2*R*, 6*R*)-hydroxynorketamine (HNK) as the main active component of ketamine metabolism responsible for the antidepressant actions of ketamine without generating the psychotomimetic side effects (Zanos et al., 2016). Conversely, Yamaguchi et al. (2018) provided evidence that blocking the formation of HNK has no effect on ketamine’s antidepressant activity in a rodent model (2018). While the clinical efficacy of HNK as a rapid-acting antidepressant has yet to be confirmed, its lack of effects at antidepressant-relevant concentrations (∼10 μM) on NMDA receptors has challenged the prevailing hypothesis that ketamine’s antidepressant actions are dependent on NMDA receptor blockade. It should be noted, however, (2R, 6R)-HNK at 50 μM does block synaptic NMDAR function (Suzuki et al., 2017), indicating the complexity of the mechanism of ketamine actions and again highlighting its critical dosage dependence. The recent FDA approval of S-ketamine (esketamine), in conjunction with an oral antidepressant, for TRD argues against a critical role of (2R, 6R)-HNK in ketamine’s antidepressant activity. However, the caveat remains that (R)-ketamine and (S)-ketamine have higher antidepressant efficacies in rodents and humans, respectively, Fukumoto et al. (2017). Several other candidates have been proposed as regulators of ketamine’s antidepressant effects including HCN channels and nicotinic receptors (Zorumski et al., 2016). A recent study by Casarotto et al. (2021) showed ketamine binds directly to the BDNF receptor TrkB and facilitates BDNF signaling, leading to restoration of synaptic function. Recent work has also indicated the opioid system plays a necessary role in ketamine’s RAAD effects, where blocking its function ameliorates ketamine’s RAAD effects in both rodents and humans (Williams et al., 2019; Klein et al., 2020). Whether this is the result of a direct or indirect interaction of ketamine remains unclear. The degree of ketamine’s interaction with these varied targets is concentration dependent, making the consideration of dosage critical in experimental design (Bonaventura et al., 2021).

Our observations that the clinically relevant dose of ketamine (2 μM; producing a tissue concentration of 5–6 μM) was not effective in blocking synaptic NMDA responses nor sufficient to induce synaptic plasticity in acute brain slices appears to raise additional doubts as to whether synaptic NMDAR inhibition should be considered as the sole underlying mechanism of ketamine’s antidepressant effect.

However, several limitations are associated with this study. First, current techniques don’t allow direct measurement of the ketamine concentration at synapses. The estimated concentration of tissue ketamine, whether *in vivo* or *in vitro*, represents an average level of ketamine across all subcellular compartments and cell types present. Secondly, NMDA receptors located on inhibitory vs. excitatory neurons may confer different sensitivity to ketamine concentrations. Field recording of NMDA responses cannot distinguish between these two neuron populations. Lastly, our electrophysiological recordings were conducted only in the hippocampal region. We have no evidence that NMDA receptors expressed in other key brain regions involved ketamine’s antidepressant action, such as PFC, exhibit the same pharmacokinetic properties as in the hippocampus.

In conclusion, ketamine is highly soluble in both water and lipid, quickly equilibrating in lipid-rich brain tissue at concentrations up to 2.8 times higher than the surrounding aCSF. Due to the concentration-dependent nature through which ketamine exerts its differential actions, these diffusion properties should be considered when designing *in vitro* studies related to the actions of ketamine. Armed with information derived from our model, it will be possible to design experiments *in vitro* which can be tailored to appropriately match the conditions that produce desired behaviors *in vivo.* This will allow for more focused studies of the relevant mechanisms of action of ketamine and, overall, a better understanding of the neurological and biochemical pathways involved in health and disease.

## Data Availability Statement

The raw data supporting the conclusions of this article will be made available by the authors, without undue reservation.

## Ethics Statement

The animal study was reviewed and approved by Des Moines University Institutional Animal Care and Use Committee.

## Author Contributions

L-LY, ZG, and BV: research design. ZG, BV, ZL, AH, KB, and L-LY: conducted experiments. ZG, BV, ZL, AH, KB, and L-LY: data analysis. ZG and L-LY: figure design and editing. ZG, LW-P, and L-LY: manuscript preparation and editing. All authors have given final approval of the version to be published.

## Conflict of Interest

The authors declare that the research was conducted in the absence of any commercial or financial relationships that could be construed as a potential conflict of interest.
